# Uncertainty quantification in coupled wildfire–atmosphere simulations at scale

**DOI:** 10.1093/pnasnexus/pgae554

**Published:** 2024-12-10

**Authors:** Paul Schwerdtner, Frederick Law, Qing Wang, Cenk Gazen, Yi-Fan Chen, Matthias Ihme, Benjamin Peherstorfer

**Affiliations:** Courant Institute of Mathematical Sciences, New York University, 251 Mercer Street, New York, NY 10012, USA; Courant Institute of Mathematical Sciences, New York University, 251 Mercer Street, New York, NY 10012, USA; Google Research, Mountain View, CA 94043, USA; Google Research, Mountain View, CA 94043, USA; Google Research, Mountain View, CA 94043, USA; Google Research, Mountain View, CA 94043, USA; Department of Mechanical Engineering, Stanford University, Stanford, CA 94305, USA; Courant Institute of Mathematical Sciences, New York University, 251 Mercer Street, New York, NY 10012, USA

**Keywords:** uncertainty quantification, multifidelity methods, neural networks, surrogate modeling, wildfire simulations

## Abstract

Uncertainties in wildfire simulations pose a major challenge for making decisions about fire management, mitigation, and evacuations. However, ensemble calculations to quantify uncertainties are prohibitively expensive with high-fidelity models that are needed to capture today’s ever-more intense and severe wildfires. This work shows that surrogate models trained on related data enable scaling multifidelity uncertainty quantification to high-fidelity wildfire simulations of unprecedented scale with billions of degrees of freedom. The key insight is that correlation is all that matters while bias is irrelevant for speeding up uncertainty quantification when surrogate models are combined with high-fidelity models in multifidelity approaches. This allows the surrogate models to be trained on abundantly available or cheaply generated related data samples that can be strongly biased as long as they are correlated to predictions of high-fidelity simulations. Numerical results with scenarios of the Tubbs 2017 wildfire demonstrate that surrogate models trained on related data make multifidelity uncertainty quantification in large-scale wildfire simulations practical by reducing the training time by several orders of magnitude from 3 months to under 3 h and predicting the burned area at least twice as accurately compared with using high-fidelity simulations alone for a fixed computational budget. More generally, the results suggest that leveraging related data can greatly extend the scope of surrogate modeling, potentially benefiting other fields that require uncertainty quantification in computationally expensive high-fidelity simulations.

**Significance Statement**

Today’s wildfires increasingly spread into populated areas, putting millions of homes at risk and impacting air quality. Numerical wildfire simulations are key building blocks for risk assessment and fire management, but uncertainties from environmental measurements must be quantified to establish trust for making high-consequence decisions. This work shows that surrogate models trained on related data make tractable uncertainty quantification with high-fidelity wildfire simulations of unprecedented scale with billions of degrees of freedom, which offers opportunities for using more complex wildfire models that can better aid fire management and evacuation planning. More generally, leveraging related data for surrogate modeling can be applied across other fields that require uncertainty quantification in large-scale simulations, such as climate modeling, aerospace engineering, and plasma physics.

## Introduction

Today’s wildfires grow faster, burn hotter, and spread more often into populated areas than ever before (1, 2). While numerical simulations are key for developing more efficient warning and prediction systems (3, 4), they are affected by large uncertainties that can pose a major challenge for making decisions about fire management, mitigation, and evacuations (5, 6). In particular, the environmental conditions such as fuel load and wind condition that are given as inputs to wildfire simulations are a major source of uncertainty due to measurement inaccuracies and sparse or incomplete data (7). It, therefore, is essential to equip numerical wildfire predictions with mean estimates, standard deviations, and sensitivities that account for input uncertainties so that decision-makers can decide how much they trust the predictions and act accordingly. However, uncertainty quantification in wildfire simulations is challenging (8). High-fidelity wildfire models that include fluid dynamics with combustion models to capture the fire–atmosphere interactions are computationally demanding (9–12), which means that even small ensemble sizes for uncertainty quantification can lead to infeasible compute runtimes (13). This has led to a use of gross simplifications in operational models that limit predictive capabilities (6, 14).

### Surrogate models trained on related data

**Fig. 1.**
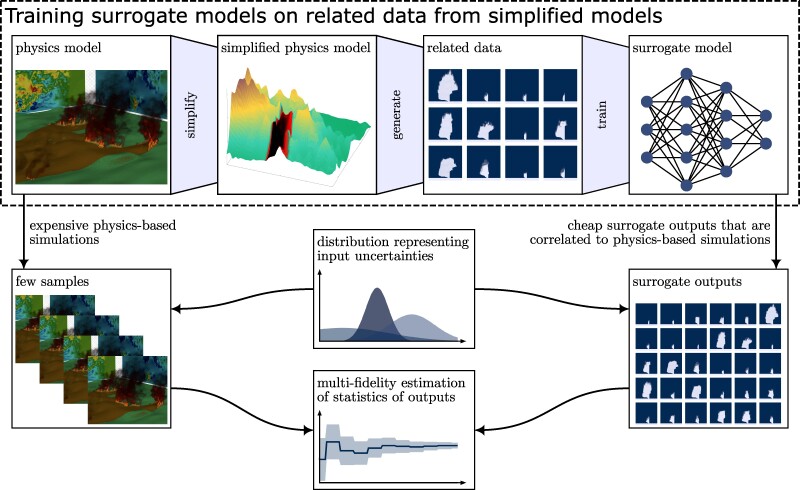
Training surrogate models on related data for multifidelity uncertainty quantification: (i) Simplifications are made to the physics model such as formulating it over smaller domains, ignoring some phenomena, linearizing, and stopping iterative numerical solvers early. (ii) The corresponding simplified model is simulated many times to rapidly generate large volumes of outputs that form the related training data. (iii) A surrogate model is trained on the related data from the simplified model. (iv) For realizations of the uncertain inputs such as environmental conditions, a few output samples are computed with the expensive physics model and many output samples are obtained with the affordable surrogate model. (v) The samples from physics and surrogate model are combined into unbiased multifidelity estimators of expectations and variances of the quantities of interest.

In this work, we scale uncertainty quantification to high-fidelity coupled fire–atmosphere simulations with billions of degrees of freedom to accurately estimate mean quantities of interest from ensembles of fire simulations. We propose an approach that trains surrogate models on related data, which are often abundantly available from previous, related physics simulations or can be cheaply generated via simplified models obtained by ignoring some of the physical phenomena, linearizing dynamics, or stopping iterative solvers early (15), see Fig. 1. We contrast related data to direct data that correspond to actual outputs or reanalysis quantities of the high-fidelity numerical simulations, which are used for training in traditional surrogate modeling but are typically prohibitively expensive to generated in many cases (16–19). We also contribute a mathematical analysis that provides a foundation for our approach and insight into the performance of the approach with respect to the quality of the related data. We demonstrate the approach on a fire scenario resembling the Tubbs 2017 wildfire, where related data are generated with a down-scaled numerical simulation in 3 h, whereas generating the same amount of direct data with the high-fidelity model would require 3 months, and thus is intractable.

Surrogate models trained on related data provide outputs that generally are not predictive about high-fidelity simulations because related data samples typically have a large bias with respect to outputs of the high-fidelity simulations. Thus, also the surrogate models will have a large bias. The key insight is that related data samples are often still correlated and that correlation is all that matters while bias is irrelevant for speeding up uncertainty quantification when surrogate models are combined with high-fidelity models in multifidelity approaches (15, 20). Multifidelity uncertainty quantification methods leverage surrogate models to accelerate the estimation of uncertainties while, in a rigorous manner, occasionally utilize expensive high-fidelity models to establish unbiased uncertainty estimators. Mathematically, we focus on the correlation that is captured by the Pearson moment correlation coefficient (21), because it is useful in multifidelity uncertainty quantification (15). This means that the outputs of our surrogate models and the wildfire simulations show similar responses to changes in inputs even though the bias can be high in the sense that the absolute values of the outputs can be vastly different. For example, increasing wind speed means faster fire spread, higher fuel density leads to more heat release; such trends are captured at least approximately by surrogate models trained on related data with data samples that are correlated to the high-fidelity simulation outputs. In contrast, traditional surrogate modeling that learns from direct data aims to accurately approximate the actual fire spread and the actual fire temperature rather than just how the fire spread and temperature change with respect to inputs.

### Literature review

Uncertainty quantification has been extensively studied for wildfire simulations; however, due to computational costs, only empirical or heuristic fire models are used (6, 14). There is work on using multilevel methods (22) but we consider orders of magnitude larger wildfire models in terms of number of degrees of freedom. Furthermore, the previous works rely either on coarse-grid approximations that provide limited speedup or traditional surrogate models that aim to keep the bias low and so require large amounts of direct training data.

While machine learning and artificial intelligence have a major impact on surrogate modeling (17, 23–34), generating sufficient direct training data samples can require many computationally expensive physics-based simulations, which is often intractable. This is one reason why there is an increasing body of literature (35–41) on training surrogate models on multifidelity and related data; however, there the aim is to use related data to improve the training of traditional surrogate models that aim to keep the bias low, which is in contrast to the approach proposed here that accepts bias as long as surrogate-model outputs are correlated, because the surrogate models are used in conjunction with multifidelity methods (20). Analogously, surrogate models based on concepts of transfer learning and one-shot learning (42, 43) aim to rapidly fine-tune a pretrained model on a new task with just a few, direct (i.e. labeled) data points. None of these studies realizes the potential of surrogate models that are trained purely on related data and thus are only correlated while having a large bias. Another line of work aims to construct surrogate models explicitly for use in multifidelity methods (44, 45), which can require less training data than generic methods; however, the training data are still direct in the sense that they correspond to input–output pairs computed with high-fidelity, physics-based models rather than related and biased data as we use in this work. Yet another line of work aims to scale surrogate modeling to large-scale settings with little training data by first performing a sensitivity analysis and sub-selecting only a few input components over which surrogate models are trained (46, 47); however, such an approach requires conducting potentially expensive sensitivity analyses first.

## Surrogate models from related data for multifidelity uncertainty quantification at scale

### Physics models with stochastic inputs

We denote a physics model as a function f:X→Y that maps an input vector x∈X, which consists of environmental conditions and model parameters, onto an output vector y=f(x)∈Y, which consists of quantities of interest that are computed from the simulation output such as the burned area. Evaluating the function *f* means performing a numerical simulation that typically incurs high computational costs, which we denote as $c_{f}>0$. Notice that we consider situations where the challenge lies with the high computational costs of evaluating *f*, rather than the high dimension of the inputs and outputs. In fact, we consider situations where there is only a low number of inputs and outputs, which is common in many science and engineering applications.

To account for incomplete knowledge and uncertain environmental conditions, we consider random input vectors *X* that follow a distribution that models input uncertainties. For example, measuring the wind speed is affected by measurement errors and noise due to data sparsity in spatial coverage and temporal resolution. In some cases, inputs have to be first obtained from upstream simulations or inverse problems, which are also affected by uncertainties that need to be carried forward. Consequently, instead of computing a deterministic output *y*, we are interested in estimating statistics of the outputs such as expected outputs E[f(X)] and variances. Notice that only the inputs are stochastic whereas the map *f* corresponding to the numerical simulation is deterministic. In the following, we omit *X* when denoting expected values E[f], variances, and correlation coefficients because all of them are taken with respect to the distribution of the inputs *X*.

A classical approach for estimating statistics is via ensembles of *m* independent and identically distributed (i.i.d.) samples $x_{1},\ldots ,x_{m}$ of the random input vector *X* and the corresponding ensemble of *m* output samples $y_{1}=f(x_{1}),\ldots ,y_{m}=f(x_{m})$. The expected output E[f] is then estimated via Monte Carlo estimation as


(1) $${\bar{y}}_{m}^{\left(f\right)}=\frac{1}{m}\sum_{i=1}^{m}f(x_{i}).$$

Obtaining an accurate estimate in Eq. (1) is challenging: Recall that each evaluation of *f* entails a numerical simulation, which can be expensive and thus severely limits the ensemble size *m* that is tractable.

### Surrogate models and multifidelity uncertainty quantification

Surrogate models provide only approximations of the outputs computed by the high-fidelity physics model *f* but often with orders of magnitude lower runtimes, see (16–19). We denote a surrogate model as $g_{\theta }\;:\;X\to Y$, which depends on a parameter vector $\theta \in R^{p}$ such as the weights of a neural network. The costs of evaluating the surrogate model are denoted as $c_{g}$ and are lower $c_{g}\ll c_{f}$ than the costs $c_{f}$ of performing a physics-based simulation with *f*. Using the surrogate $g_{\theta }$ instead of the high-fidelity physics model *f* in the Monte Carlo estimator in Eq. (1) leads to speedups, but it also means that the estimator is biased with respect to E[f] because the surrogate model provides an approximation of the outputs of *f* only. To leverage the surrogate model for achieving runtime speedups while avoiding the introduction of a bias, we use the surrogate model $g_{\theta }$ in a multifidelity Monte Carlo estimator (15). The multifidelity Monte Carlo estimator of E[f] is based on a variance reduction technique called control variates (48) and is given by


(2) $${\bar{y}}_{m_{1},m_{2}}={\bar{y}}_{m_{1}}^{\left(f\right)}+\alpha ({\bar{y}}_{m_{2}}^{\left(g\right)}-{\bar{y}}_{m_{1}}^{\left(g\right)}),$$

where ${\bar{y}}_{m_{1}}^{\left(f\right)}$ is a Monte Carlo estimator as in Eq. (1) based on $m_{1}$ samples from *f*, and ${\bar{y}}_{m_{1}}^{\left(g\right)},{\bar{y}}_{m_{2}}^{\left(g\right)}$ are Monte Carlo estimators based on $m_{1}$ and $m_{2}$ samples, respectively, of the surrogate model $g_{\theta }$; details in the Supplementary material. The number of samples $m_{1}$ and $m_{2}$ as well as the coefficient *α* in the estimator in Eq. (2) can be chosen optimally to minimize the mean-squared error (MSE) of ${\bar{y}}_{m_{1},m_{2}}$. We remark that similar multifidelity estimators based on control variates have been introduced for higher-order moments and sensitivity analyses (49), to which our approach would also be applicable.

Using a multifidelity estimator as given in Eq. (2) is key for our approach of training surrogate models on related data: First notice that ${\bar{y}}_{m_{1},m_{2}}$ is unbiased in the sense that $E[{\bar{y}}_{m_{1},m_{2}}]=E[f]$, independent of the bias of the surrogate model


(3) $$b(g_{\theta })=|E[g_{\theta }]-E[f]|$$

compared with the physics model *f*. One can see the unbiasedness of the multifidelity estimator by noting that the expected value $E[{\bar{y}}_{m_{2}}^{\left(g\right)}-{\bar{y}}_{m_{1}}^{\left(g\right)}]$ of the difference term in Eq. (2) is zero and thus vanishes, which means that the expected value of the multifidelity estimator is $E[{\bar{y}}_{m_{1},m_{2}}]=E[{\bar{y}}_{m_{1}}^{\left(f\right)}]=E[f]$ and thus ${\bar{y}}_{m_{1},m_{2}}$ is an unbiased estimator of E[f]. Thus, even if the surrogate model $g_{\theta }$ has a large bias in the sense of Eq. (3), the multifidelity estimator remains unbiased. Second, we want to understand the MSE $e({\bar{y}}_{m_{1},m_{2}})$ of the estimator in Eq. (2). Because we already know that the multifidelity estimator is unbiased, the MSE of the estimator equals the variance of the estimator, $e({\bar{y}}_{m_{1},m_{2}})=V[{\bar{y}}_{m_{1},m_{2}}]$. We now need a few more quantities to understand and interpret the MSE of ${\bar{y}}_{m_{1},m_{2}}$. Let $\sigma_{f}=\sqrt{V[f]}$ and $\sigma_{g}=\sqrt{V[g_{\theta }]}$ denote the standard deviations of *f* and $g_{\theta }$, respectively, with respect to the random input vector *X*. Let further


(4) $$\rho (f,g_{\theta })=\frac{\text{Cov}[f,g_{\theta }]}{\sigma_{f}\sigma_{g}},$$

denote the Pearson-moment correlation coefficient between the outputs of the physics model *f* and the surrogate model $g_{\theta }$ (21). The operator Cov denotes the covariance. Setting $\alpha =\sigma_{f}\sigma_{g}^{-1}\rho (f,g_{\theta })$ in the estimator in Eq. (2), which is the optimal choice that minimizes the variance of the estimator (15), applying transformations based on properties of the variance and leveraging that the samples $x_{1},\ldots ,x_{m}$ of the random input vector are independent, one obtains from Eq. (2) that the MSE of the estimator is


(5) $$e({\bar{y}}_{m_{1},m_{2}})=\frac{\sigma_{f}^{2}}{m_{1}}-\left(\frac{1}{m_{1}}-\frac{1}{m_{2}}\right)\sigma_{f}^{2}\rho (f,g_{\theta }{)}^{2}.$$

A full derivation of the MSE shown in Eq. (5) as well as the optimal choice for the samples $m_{1}$ and $m_{2}$ can be found in (15) and the Supplementary material.

The key quantity for interpreting the MSE is the correlation coefficient $\rho (f,g_{\theta })$. The higher the squared correlation coefficient $\rho (f,g_{\theta })$, the lower the MSE $e({\bar{y}}_{m_{1},m_{2}})$. Notice that the term $\frac{1}{m_{1}}-\frac{1}{m_{2}}\geq 0$ is nonnegative because naturally more samples $m_{1}\leq m_{2}$ are taken from the cheap surrogate model, see (15) for details about the optimal choice of $m_{1}$ and $m_{2}$. It is critical that the MSE given in Eq. (5) depends on the correlation coefficient given in Eq. (4) only; and not on the bias given in Eq. (3) of the surrogate model. Thus, for the surrogate model $g_{\theta }$ to be effective in estimating the expected value with the multifidelity estimator given in Eq. (2), it is sufficient that the correlation between the physics model *f* and the surrogate model $g_{\theta }$ is high.

Let us briefly remark on the coefficient *α* in the estimator in Eq. (2). The coefficient *α* is a weight of the difference term that includes the surrogate model in the multifidelity estimator. We used the optimal $\alpha =\sigma_{f}\sigma_{g}^{-1}\rho (f,g_{\theta })$ in Eq. (2) to derive the MSE given in Eq. (5), where optimal means that it minimizes the variance (15). Intuitively, setting $\alpha =\sigma_{f}\sigma_{g}^{-1}\rho (f,g_{\theta })$ means that it weights samples from the surrogate model proportionally to the correlation coefficient and inverse proportionally to the variance. Thus, broadly speaking, a higher correlated surrogate model with low variance leads to a higher weight because the surrogate model can be trusted more than when the correlation is low or the variance of the surrogate model is high.

We further remark that the MSE given in Eq. (5) treats the surrogate model $g_{\theta }$ as deterministic and thus ignores potential variations in the surrogate model due to stochastic training and random initializations, which is common when surrogate models are based on neural networks. We discuss this point in more detail in the Supplementary material, where we show that the variations introduced by different random initializations of the training are small compared with the uncertainties introduced by the random input vector *X* in our numerical experiments.

### Training on related data

We now exploit that the MSE given in Eq. (5) of the estimator given in Eq. (2) depends on the correlation coefficient $\rho (f,g_{\theta })$ between the surrogate model $g_{\theta }$ and *f*, and not on the bias given in Eq. (3). Thus, high correlation between $g_{\theta }$ and *f* is sufficient for achieving a low MSE, whereas providing good approximations of the outputs of *f* in the sense of the bias defined in Eq. (3), i.e. a relative/absolute error, is unnecessary.

Requiring a surrogate model to have a high correlation is often a weaker requirement than having a low bias: Only the trend of the surrogate model $g_{\theta }$ and the physics model *f* have to be similar. In particular, it suffices to train $g_{\theta }$ on related data $D=\{(x_{i},h(x_{i})){\}}_{i=1}^{N}$ generated from a data source h:X→Y that is related to the physics model in the sense that the correlation ρ(f,h) is high. There are often plenty of related data samples available or they can be cheaply generated from related data sources, even in data-scarce science and engineering applications. For example, in our fire simulation application, we cheaply generate related data by running fire simulations on smaller scales obtained by simply shrinking the spatial domains, which leads to fewer degrees of freedom and thus lower runtimes per data sample. While the outputs obtained with the fire simulations on the reduced domains are not accurately approximating the outputs obtained with the high-fidelity, large-scale simulations in the sense of the bias defined in Eq. (3), the outputs are correlated to the outputs of the high-fidelity simulations in the sense of the correlation coefficient given in Eq. (4), see below. Other strategies for generating related data points rely on simplified physics models that ignore some of the phenomena, build on linear approximations, or stop iterative solvers early (20).

Motivated by this, we propose to train the surrogate model $g_{\theta }$ on related training data D so that the trained surrogate model $g_{\theta }$ yields outputs that are correlated with outputs of *f*: We formally have the correlation ρ(f,h) between the physics model outputs and the data source *h* and the correlation $\rho (h,g_{\theta })$ between the data source *h* and the surrogate model $g_{\theta }$. The following proposition provides upper and lower bounds on the correlation coefficient $\rho (f,g_{\theta })$ between the physics model and the surrogate model. A proof of the proposition can be found in the Supplementary material.

**Proposition 1.** *The correlation coefficient $\rho (f,g_{\theta })$ between outputs of the physics model *f* and the surrogate model $g_{\theta }$ is bounded from above and below as*

$$\begin{array}{rl} \rho (f,g_{\theta }) & \leq \rho (f,h)\rho (h,g_{\theta })+\sqrt{\left(1-\rho (f,h{)}^{2})(1-\rho (h,g_{\theta }{)}^{2}\right)},\\ \rho (f,g_{\theta }) & \geq \rho (f,h)\rho (h,g_{\theta })-\sqrt{\left(1-\rho (f,h{)}^{2})(1-\rho (h,g_{\theta }{)}^{2}\right)}. \end{array}$$

**Fig. 2.**
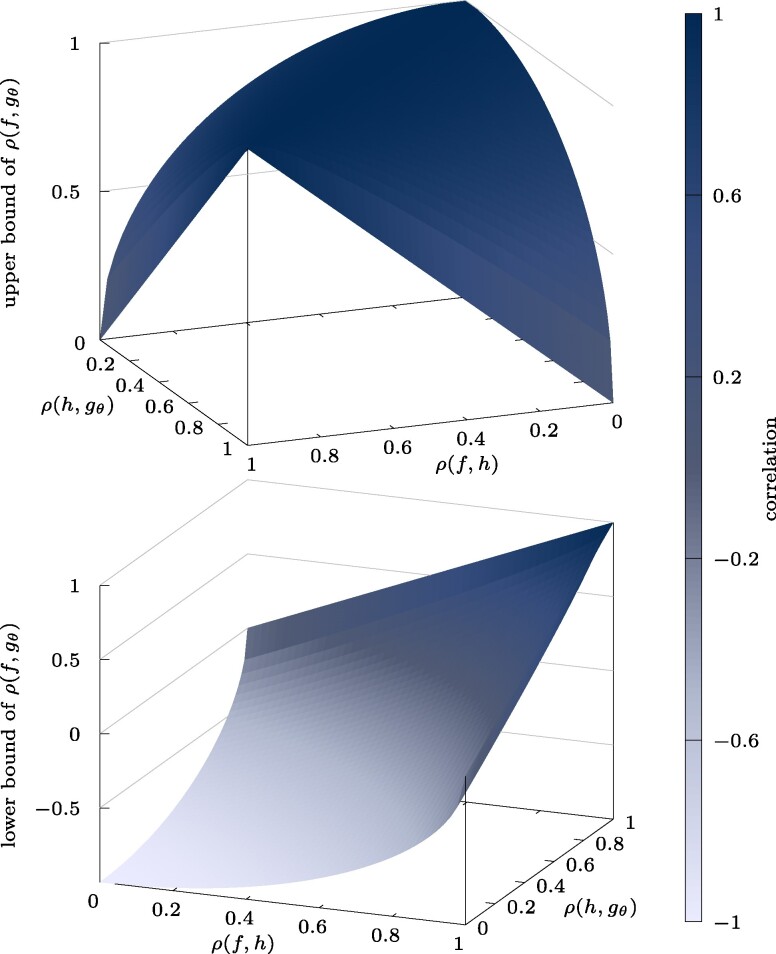
The plots visualize the upper and lower bound (Proposition 1) of the correlation between the surrogate model $g_{\theta }$ and the physics model *f* when the surrogate model $g_{\theta }$ is trained on a related data source *h* that is correlated to *f*. The upper and lower bounds depend on the correlation between the data source *h* and the physics model *f* (“quality of the data”) as well as correlation between the data source *h* and the surrogate model $g_{\theta }$ (“quality of training”).

The upper bound is maximized when ρ(f,h) and $\rho (h,g_{\theta })$ are balanced, which indicates that if *f* and *h* are poorly correlated, then it is unnecessary to accurately train $g_{\theta }$ to match well the correlated data sampled from *h*. In Fig. 2, we plot the upper bound of $\rho (f,g_{\theta })$ against ρ(f,h) and $\rho (h,g_{\theta })$, which visualizes that the upper bound is high when ρ(f,h) and $\rho (h,g_{\theta })$ are of comparable magnitude, i.e. when they are balanced. With regards to the lower bound, which is also shown in Fig. 2, note that the squared correlation coefficient $\rho (f,g_{\theta }{)}^{2}$ enters in the MSE in Eq. (5) rather than directly $\rho (f,g_{\theta })$. The lower bound stated in Proposition 1 shows that the squared correlation coefficient $\rho (f,g_{\theta }{)}^{2}>0$ is greater than zero if


(6) $$\rho (f,h{)}^{2}+\rho (h,g_{\theta }{)}^{2}>1$$

holds, see the Supplementary material for a detailed derivation. This result implies that both the high-fidelity model *f* and the data source *h* as well as the data source *h* and the surrogate model $g_{\theta }$ need to be sufficiently well correlated so that the correlation between the high-fidelity model and the surrogate model is not zero. We will show in the numerical experiments in Fig. 4 that this condition is met with a large margin in all of our experiments. Further visualizations of the bounds and the condition given in Eq. (6) can be found in the Supplementary material.

Critically, as long as the squared correlation coefficient between $g_{\theta }$ and *f* is high, the surrogate model $g_{\theta }$ is useful in multifidelity estimation as with the multifidelity Monte Carlo estimator given in Eq. (2).

## Surrogate models from related data for Tubbs 2017 wildfire scenarios

### Coupled fire–atmosphere model with about eight billion degrees of freedom

**Fig. 3.**
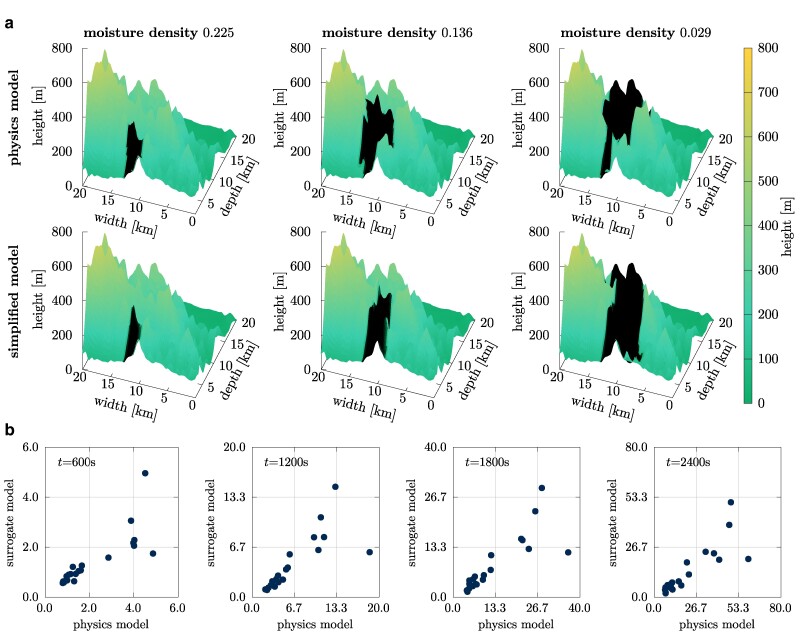
The high-fidelity physics model as well as the simplified model predict that increasing moisture density leads to smaller burned areas, which indicates that the simplified model captures the trends of the high-fidelity physics model and thus is sufficient for generating related training data in this example. a) Burned area in Tubbs domain for three input realizations with moisture densities 0.225, 0.136, and 0.029. The surface plots show the terrain height using a color gradient and the burned area in black. b) Comparison of burned areas over 20 different input realizations (environmental configurations) at different times after ignition between the large-scale simulation and the surrogate model trained on related data.

The wildfire scenarios that we consider are motivated by the Tubbs wildfire that took place in Northern California in October 2017 (50, 51). We simulate the spread of a wildfire on a 20 km by 20 km area with a terrain that is representative of the mountain area between Calistoga and Santa Rosa, see Fig. 3. The fire is simulated up to heights of 4 km to accurately capture atmospheric effects. The domain is resolved with 20 m in horizontal and 4 m in vertical direction, which corresponds to 1,024×1,024×1,024 grid points and leads to about eight billion degrees of freedom because the physics model is formulated over density, velocity in the three spatial directions, potential temperature, oxygen mass fraction, solid temperature, and fuel density. The physics model is based on the Navier–Stokes equations in a low-Mach formulation (11), which eliminates the constraints on the time step size by the acoustic waves in the numerical simulation. A multiphase combustion model is used to represent the fire behavior, and it is fully coupled with the fluid dynamics of the atmosphere to capture the fire–atmosphere interactions (12). We remark that the model has been validated with other, controlled fire scenarios in (7). Details about the model can be found in the Supplementary material.

The input vector *X* to the simulation contains the wind speed, initial fuel density, and initial moisture content, which are key environmental conditions that influence the fire dynamics. The components of the input vector *X* are considered uncertain and distributed uniformly in the intervals [5.0,12.0], [0.2,3.0], and [0.03,0.12], respectively so that


(7) X∼U([5,12]×[0.2,3]×[0.03,0.12]),

where U denotes a uniform distribution. The output is the burned area over time after ignition. The burned area is computed via the fuel density field to identify areas where fuel density has decreased, which indicates burning; details about computing the burned area can be found in Supplementary material. For a single realization of the random input vector, the simulation (one evaluation of *f*) takes about 19.5 h on 128 Tensor Processing Unit (TPU) v5e cores of Google Cloud. Even when performing ten simulations in parallel, generating 1,000 direct data points for training surrogate models would take almost 3 months on 1,280 TPUs, which is intractable. In our experiments below, we have available up to 20 simulation results from the high-fidelity physics model, which is too little for training surrogate models. These 20 simulations are also insufficient for estimating moments with quadrature rules on grids, because even only five grid points in each dimension would already require >120 simulations in our case and thus six times the amount of simulations that we have available.

### Wildfire simulations: Related data source

For training surrogate models, we generate related data by using a small-scale simulation, in which the domain is scaled (shrunken) down by a factor of 10 proportionally. Correspondingly, we require only 256×256×160 spatial grid points so that one simulation takes on average 28 min on eight TPU cores. Generating 1,000 related training data samples takes 2.92 h with 10 simulations in parallel and each on 128 TPU cores. Thus, the runtime of generating the training data are reduced from 3 months to <3 h. We train as surrogate model $g_{\theta }$ a multilayer perceptron with three input nodes (fuel density, moisture density, and wind speed), three hidden layers of width five, and a linear output layer on the related data to obtain outputs that are correlated with the burned area predicted by the physics model; details of the training setup can be found in the Supplementary material.

**Fig. 4.**
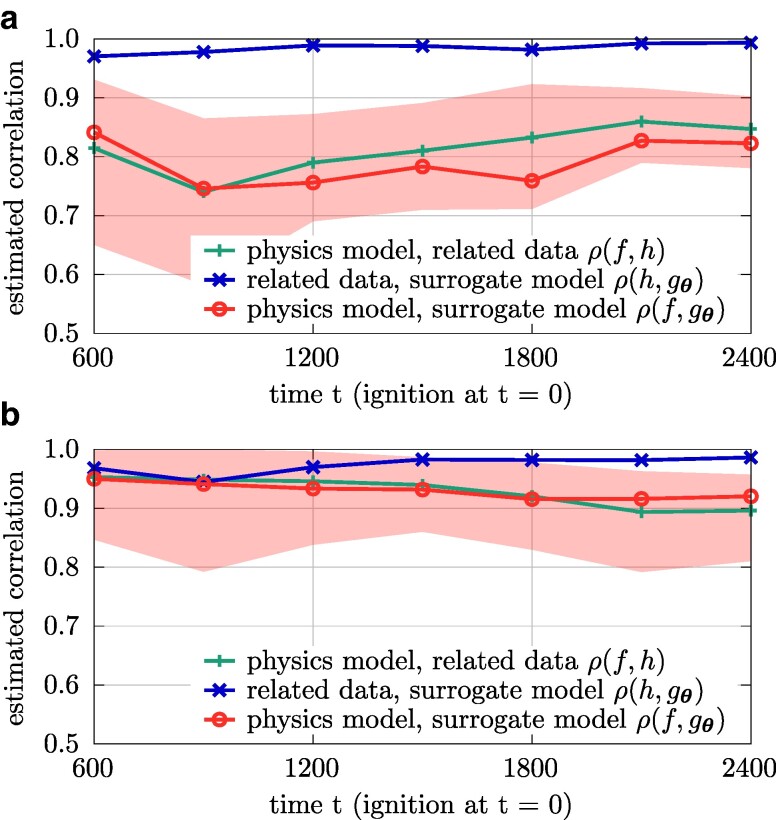
The estimated correlation coefficients show that the physics model is well correlated to the surrogate models trained on the related training data. The shaded area corresponds to the upper and lower bound of the correlation coefficient $\rho (f,g_{\theta })$, see Proposition 1. a) Input distribution given in Eq. (7). b) Input distribution given in Eq. (8).

To demonstrate the correlation between the high-fidelity physics and the simplified model, we plot in Fig. 3a the burned area with respect to a decreasing moisture density. The absolute value of the burned area given by the physics model and the simplified model differ by >30%, which indicates a large bias in the sense of Eq. (3). However, with both models we obtain that the burned area increases with decreasing moisture density, which indicates that the outputs of the physics-based and simplified model are correlated and the simplified model is able to reproduce a physically meaningful behavior. Figure 3b shows the correlation more directly by plotting the outputs of the surrogate model obtained from training data generated with the simplified model against the predictions of the physics model over time *t* when varying the input components. The estimated correlation coefficient given in Eq. (4) is shown in Fig. 4a, with the mean value of the correlation coefficient being 0.8335 over time *t*.

### Wildfire simulations: Performance

We now use the surrogate model trained on related data in the multifidelity estimator given in Eq. (2) to estimate the expected burned area. We compare three cases. First, we consider the physics model alone in a regular Monte Carlo estimator given in Eq. (1), which is expensive but leads to an unbiased estimator. Second, we use the surrogate model alone in a regular Monte Carlo estimator, which leads to a biased estimator because the surrogate model has a bias with respect to the physics model. Third, we leverage the surrogate model in a multifidelity Monte Carlo estimator given in Eq. (2) together with the physics model, which provides an unbiased estimator. We stress that the multifidelity estimator of the burned area is unbiased even though the surrogate model is used and has been trained on related data rather than on direct data.

**Fig. 5.**
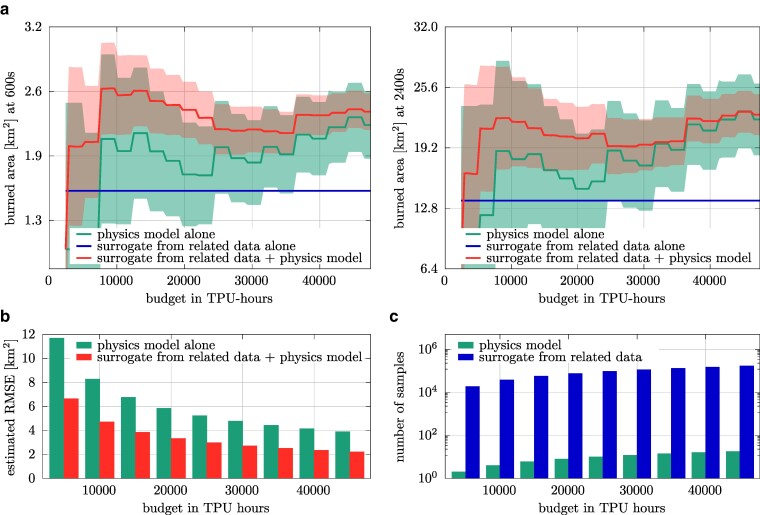
Scenario with input distribution given in Eq. (7). a) Using the surrogate models trained on related data together with the physics models leads to unbiased multifidelity estimators of the expected burned area that exhibit less variance with increasing computational budget (TPU hours) than using the physics model alone. Including the surrogate model from related data achieves accurate estimates of the expected burned area at already around 10,000 TPU hours, whereas using the physics model alone requires up to almost 50,000 TPU hours to achieve a comparable expected burned area. For a comparison at additional times after ignition; see Fig. S3a. b) Estimates of the RMSEs are in agreement with the previous results and indicate that including the surrogate models trained on related data leads to almost 2× more accurate estimates of the expected burned area compared with using the physics model alone. c) The plot shows the number of samples used from the physics and the surrogate model when the two models are combined by the multifidelity estimator.

The estimated expected burned area is shown in Fig. 5a, together with the estimated root mean squared error (RMSE) shown as shaded area. Notice the bias obtained when the surrogate model is used alone, which is in agreement with the discussion above that a surrogate model trained on related data is *not* predictive in the sense that the surrogate model outputs can have a large bias in the sense of Eq. (3) and thus the surrogate model cannot replace the physics models. However, the surrogate model is still useful, namely when it is combined with the physics model within the multifidelity estimator, where it introduces no bias. As the results in Fig. 5a show, combining the surrogate model and the physics models leads to estimates that predict a burned area of about $2.4\;{\mathrm{km}}^{2}$ at already about 10,000 TPU hours at time t=600 s, whereas the estimator that uses the physics model alone takes almost 50,000 TPU hours to converge to a comparable burned area. Similar observations hold at later times after ignition. Figure 5b shows the estimated RMSEs of the estimators as a bar plot. We stress that the estimated RMSEs shown in Fig. 5b and the estimated RMSEs shown as shaded area in Fig. 5a are affected by estimation errors and we thus use them as crude indicators only to see if they are in agreement with the other results, see Supplementary material for how we estimate the RMSEs. In agreement with the low variance of the curve corresponding to the estimator obtained with the surrogate model together with the physics model shown in Fig. 5a, the estimated RMSE indicates that using the surrogate model in the multifidelity estimator leads to a lower RMSE than using the samples from the physics model alone. The numbers of samples used from the physics model versus the correlated surrogate model at time t=2,400 s are shown in Fig. 5c.

### Wildfire simulations: Performance with reused surrogate model

It is common to train a surrogate model on an input distribution with a larger variance so that it can be reused on input distributions with a lower variance in scenarios where more information is available. To illustrate this, we consider a smaller range of wind speeds and fuel densities so that


(8) X∼U([7,10.25]×[0.9,2.3]×[0.03,0.12]).

**Fig. 6.**
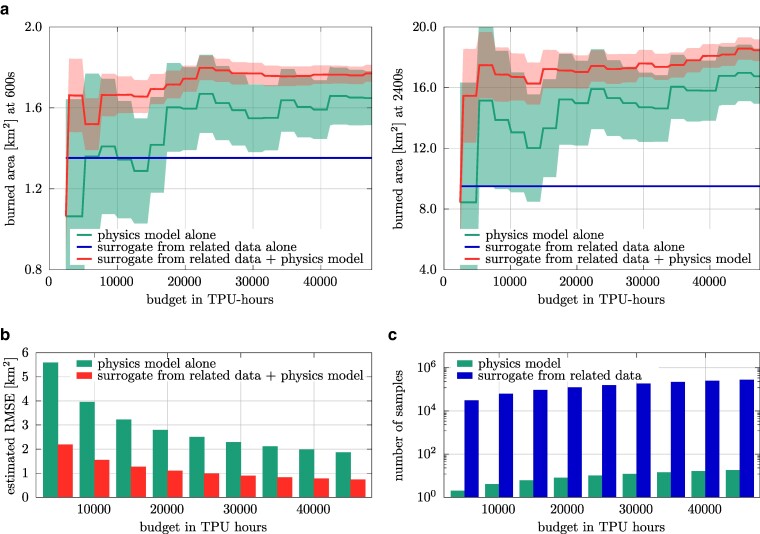
Scenario with input distribution given in Eq. (8): Already for low computational budgets of about 10,000 TPU hours, the surrogate model trained on related data together with the physics model lead to a multifidelity estimator of the expected burned area that shows little variance as the budget of TPU hours is increased. This indicates that including the surrogate model from related data provides more accurate estimates than using the physics model alone, because the surrogate model is used via a multifidelity estimator so that it introduces no bias. The estimated RMSEs are in agreement with these results and indicate an almost 3× lower RMSE than the estimator that uses the physics model alone. For a comparison at additional times after ignition, see Fig. S3b.

We reuse the surrogate model from the previous scenario and demonstrate now that it still leads to an efficient multifidelity estimator even over this changed input distribution because it is still correlated with the physics model, see Fig. 4b. The predictions of the estimators are shown in Fig. 6. The lower variance of the input distribution leads to a higher correlation of the surrogate model to the physics model, which results in a multifidelity estimator with the surrogate model that shows less variation over the budget and thus settles more quickly on a value for the estimated expected burned area compared with using the physics model alone.

## Discussion

In this study, we demonstrate that training surrogate models on related data and using them together with multifidelity estimators allows scaling uncertainty quantification to estimate expectations of quantities of interest from ensembles of large-scale wildfire simulations with billions of degrees of freedom. Our approach captures more accurately the burned area than using either surrogate models or physics models alone for the same computational costs. This study further demonstrates that viewing surrogate modeling through a broader lens than just aiming for accurate point-wise predictions of the outputs of physics models can greatly extend the scope of surrogate modeling. In particular, the results of this study show that it is sufficient for surrogate models to yield outputs that are statistically correlated with the outputs of physics models with respect to the Pearson moment correlation coefficient; it is unnecessary that the surrogate models are point-wise predictive about the quantities of interest in terms of having a low bias. This allows training surrogate models on related data rather than on direct data that directly describe the input–output relationship given by the physics model. Learning from related data broadens the scope of data-driven surrogate modeling to settings where direct data are scarce, as demonstrated by our application to wildfire simulations.

The focus of this study is on uncertainty quantification, for which surrogate models trained on related data are useful in multifidelity computations such as multifidelity Monte Carlo methods. However, we expect surrogate models that provide correlated outputs to be useful beyond our specific setting of uncertainty quantification such as performing sensitivity analyses with multifidelity estimators (49) and estimating objective functions in optimal design problems under uncertainty, inverse problems, and control. Overall, the findings of this study encourage the perspective on surrogate modeling that shifts the focus away from the classical aim of directly mimicking physics-based simulations towards being another information source in multifidelity computations (20).

## Supplementary Material

pgae554_Supplementary_Data

## Data Availability

Original data created for the study are or will be available in a persistent repository upon publication. The type of data is code and is available via a Zenodo repository https://doi.org/10.5281/zenodo.11391078.

## Supplementary Material

Supplementary materialis available at *PNAS Nexus* online.
