# Supplementary material for: Uncertainty quantification in coupled wildfire–atmosphere simulations at scale
Source: PNAS Nexus. 2024 Dec 10;3(12):pgae554. doi: 10.1093/pnasnexus/pgae554 (PMC11660924; doi:10.1093/pnasnexus/pgae554)
Supplement: pgae554_Supplementary_Data [file pgae554_supplementary_data.pdf]

PNAS Nexus, 2021, pp. 1–3

doi: DOI HERE
Advance Access Publication Date: Day Month Year
Supplementary Material

SUPPLEMENTARY MATERIAL

Uncertainty quantification in coupled wildfire-atmosphere simulations at scale

Paul Schwerdtner,[a] Frederick Law,[a] Qing Wang,[b] Cenk Gazen,[b] Yi-Fan Chen,[b] Matthias Ihme[b,c] and Benjamin Peherstorfer[a,*]

[a]Courant Institute of Mathematical Sciences, New York University, New York, NY 10012, USA, [b]Google Research, Mountain View, CA 94043, USA and [c]Department of Mechanical Engineering, Stanford University, Stanford, CA 94305, USA
[*]To whom correspondence should be addressed: Benjamin Peherstorfer, Courant Institute of Mathematical Sciences, New York University, 251 Mercer Street, New York, NY 10012, USA. Email: pehersto@cims.nyu.edu
FOR PUBLISHER ONLY Received on Date Month Year; accepted on Date Month Year

Abstract

This is the supplementary material for *Uncertainty quantification in coupled wildfire-atmosphere simulations at scale.*

Multi-fidelity Monte Carlo estimation

The multi-fidelity Monte Carlo estimator that we use has been introduced in [4] for multiple models. We consider the case of only two models; the surrogate model trained on related data and the physics model. The number of samples m_1 and m_2 as well as the coefficient α in the estimator (2) are chosen such that the variance of the estimator is minimized for a given computational budget p, which leads to $m_1^* = \frac{p}{c_f + c_g r}$, $m_2^* = rm_1^*$, $\alpha^* = \frac{\rho\sigma_f}{\sigma_g}$, where $r = \sqrt{\frac{c_f \rho^2}{c_g(1-\rho^2)}}$, as shown in [4]. In our implementation, we round down m_1^* and m_2^* to obtain integer numbers of model evaluations and to stay within the computational budget p. The MSE (5) of the multi-fidelity estimator with optimal sample allocation m_1^*, m_2^* and coefficient α for a given computational budget p is

$$e(\bar{y}_{m_1,m_2}) = \frac{\sigma_f^2(1-\rho^2)}{(m_1^*)^2 w_1} p, \tag{S1}$$

and the MSE of the regular Monte Carlo estimator is $\sigma_f^2 w_1/p$. In Figure 5 and Figure 6 we report estimates of the RMSE of the estimators. For the MFMC, we use (S1), where the quantities σ_f, σ_g, ρ are estimated via Monte Carlo on the same samples as used in the multi-fidelity estimators, which is a common approach [2]. The analogous approach is used for the RMSE of the regular Monte Carlo estimator. We stress that the RMSE is plotted only as an additional indication for the quality of the estimate obtained with the surrogate models trained on related data in Figures 5–6. It agrees with the behavior of the curves corresponding to the expected burned area, which show that the surrogate model trained on related data with the MFMC estimator leads to less variance and settles quicker on a value for the expected burned area with less TPU resources than the regular Monte Carlo estimator based on the physics model.

Analogous results to Figure 5–6 for time $t = 1200$ s and $t = 1800$ s after ignition are shown in Figure S3. The results are in agreement with the results shown in Figure 5–6.

To provide a proof for Proposition 1, we re-state it here with generic random variables.

Proposition S1. *Let X, Y, Z be real-valued random variables with bounded first and second moments. Let ρ_{xy}, ρ_{xz}, and ρ_{yz} denote the Pearson correlation coefficients between X, Y, X, Z, and Y, Z respectively. Then, for given ρ_{xy} and ρ_{yz}, we have that*

$$\rho_{xz} \leq \rho_{xy}\rho_{yz} + \sqrt{(1-\rho_{xy}^2)(1-\rho_{yz}^2)},$$
$$\rho_{xz} \geq \rho_{xy}\rho_{yz} - \sqrt{(1-\rho_{xy}^2)(1-\rho_{yz}^2)}.$$

Proof Since scaling and shifting does not change the correlation of random variables, we can assume without loss of generality that X, Y, and Z are zero-mean and unit-variance. Thus, the covariance matrix is given by

$$R = \begin{bmatrix} 1 & \rho_{xy} & \rho_{xz} \\ \rho_{xy} & 1 & \rho_{yz} \\ \rho_{xz} & \rho_{yz} & 1 \end{bmatrix}. \tag{S2}$$

© The Author 2021. Published by Oxford University Press. All rights reserved. For permissions, please e-mail: journals.permissions@oup.com

Since R is positive semi-definite, it must satisfy $\det(R) \geq 0$. Using the Laplace expansion for the computation of the determinant, this yields $1 - \rho_{yz}^2 - \rho_{xy}^2 + 2\rho_{xz}\rho_{xy}\rho_{yz} - \rho_{xz}^2 \geq 0$. Looking for values at which this inequality attains 0 leads to a quadratic polynomial in ρ_{xz}, which we solve to obtain the lower and upper bounds. □

The MSE (5) of the multi-fidelity estimator depends on the squared correlation coefficient $\rho(f, g_\theta)^2$ so that a larger value of $\rho(f, g_\theta)^2$ leads to a smaller MSE. To give a better intuition on the performance of our method, we now provide a lower bound for the squared correlation coefficient $\rho(f, g_\theta)^2$. Starting with the lower bound

$$\rho(f, g_\theta) \geq \rho(f, h)\rho(h, g_\theta) - \sqrt{(1 - \rho(f, h)^2)(1 - \rho(h, g_\theta)^2)}$$

given in Proposition 1 (and equivalently in Proposition S1), we obtain

$$\rho(f, g_\theta)^2 \geq \left(\rho(f, h)\rho(h, g_\theta) - \sqrt{(1 - \rho(f, h)^2)(1 - \rho(h, g_\theta)^2)}\right)^2$$

if

$$\rho(f, h)\rho(h, g_\theta) - \sqrt{(1 - \rho(f, h)^2)(1 - \rho(h, g_\theta)^2)} > 0 \qquad \text{(S3)}$$

holds. Furthermore, we see that $\rho(f, g_\theta)^2 > 0$ (strictly greater than zero) holds under condition (S3). Notice that condition (S3) is equivalent to the condition

$$\rho(f, h)^2 + \rho(h, g_\theta)^2 > 1 \qquad \text{(S4)}$$

given in the main text. To gain some more intuition about this result, we visualize the lower bound of $\rho(f, g_\theta)^2$ in Figure S1, which shows that $\rho(f, g_\theta)^2$ is greater than zero if (S4) is satisfied.

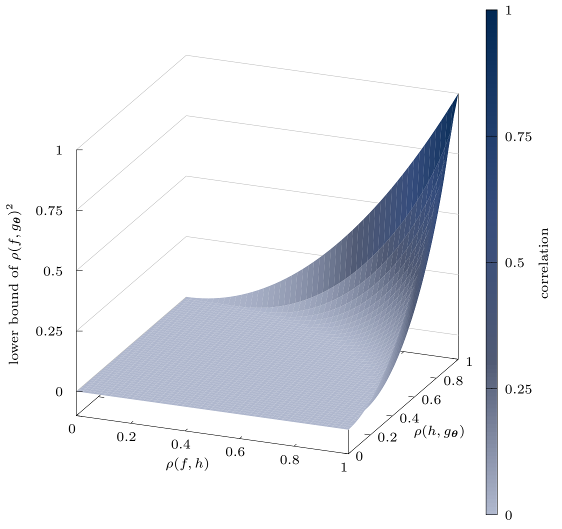

Figure S1. The plot visualizes the lower bound of the squared correlation coefficient between the surrogate model g_θ and the physics model f when the surrogate model g_θ is trained on data from a related correlated data source h. Notice that the squared correlation coefficient $\rho(f, g_\theta)^2$ is greater than zero if $\rho(f, h)^2 + \rho(h, g_\theta)^2 > 1$.

Fire simulation setup

All simulations are conducted using the TensorFlow fluid flow simulation framework SwirlLm [5], which is available at `https://github.com/google-research/swirl-lm`. In this study, we adopted the large-eddy simulation approach, where the gas phase variables are represented by the Favre-filtered conservation equations for mass, momentum, energy, and species. The processes of solid fuel pyrolysis, evaporation, and combustion are modeled with a one-step mixing-limited oxidation reaction [3]. Convective and radiative heat transfer, as well as mass and heat exchange of moisture evaporation are considered for the interaction of the fire with the ambient air [5].

The simulations consider the spread of a fire in a 20 km by 20 km area, modeled after the terrain between Calistoga and Santa Rosa. Additionally, the flow simulation considers an altitude of 4 km. An inflow-outflow boundary condition is applied on the two ends of the computational domain along the streamwise direction. The mean velocity at the inflow is prescribed by a log profile, with a superposition of turbulent fluctuations that are generated with a digital filter [1]. The two sides of the computational domain along the lateral direction and the top of the domain are treated with the free-slip condition. The bottom of the domain is represented by the terrain, which is modeled with the immersed boundary method. For the high fidelity simulation, the domain is divided into a 1024^3 grid with a total of 1,073,741,824 nodes. In time, the step-size is $t = 0.025$ s and the simulation is run for 6000 s, with ignition at 2500 s. The high-fidelity simulations are run on a TPU v5e with 128 tensor cores. The runtime for each simulation is about 19.5 hours, which leads to a cost per simulation of 2496 TPU-core-hours. For the faster small-scale simulations, the domain is scaled down proportionally, such that the small-scale simulations only consider a 2 km by 2 km area with an altitude of 200 m. The height-map is scaled down accordingly. The smaller size allows one to consider a smaller grid of size $256 \times 256 \times 160$ and to simulate only for 600 s with ignition at 250 s. Using this setup, the simulations can be carried out on a TPU v2-8 with 8 cores and require a runtime of around 28 minutes, which we parallelize ultimately on 1280 cores for parallel computation of training samples. For our multi-fidelity analysis, we shift the ignition time to $t = 0$ and scale the simulated time of the small-scale simulations up by a factor of 10.

Our quantity of interest is the burned area, which we calculate as follows: At each time t after ignition, we check the fuel density field to identify areas where fuel density has decreased, indicating burning, as fuel cannot be displaced by other means. By factoring in the terrain slope, we map these identified areas to determine the corresponding burned region. We then sum these regions to calculate the total burned area at time t.

Surrogate model training

The surrogate model $g_{\theta(t)}$ for estimating the burned area at time t after ignition is a multi-layer perceptron with three input nodes (fuel density, moisture density, and wind speed), three hidden layers of width five, one output node, and selu activation functions. Notice that for each time t a different neural network with weight vector $\theta(t)$ is trained. For each time point, we have 1000 related data samples available for training, which are split into 900 training samples and 100 validation samples. For training, we use 100,000 ADAM iterations, the MSE loss, and select the surrogate model with the lowest validation loss. Note that the validation data points correspond to different realizations

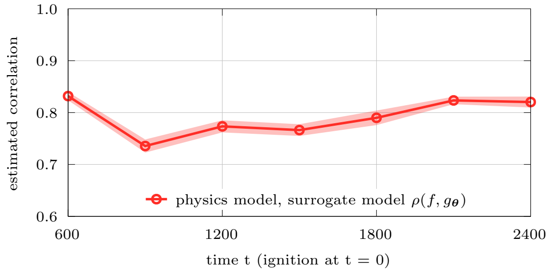

Figure S2. Means and standard deviations of the estimated correlation coefficient for different times after ignition. Statistics are computed for 20 surrogate model replicates that are trained using different random seeds. The standard deviation is visualized as shaded area about the mean value.

of the random input variables than the ones used for the actual multi-fidelity estimation and thus the surrogate model has to generalize when used in the multi-fidelity estimator. The implementation was done in JAX with the flax and optax packages. Code for training as well as the trained model can be found at `https://github.com/Algopaul/fireuq.git`. We plot the correlation coefficient $\rho(f, g_{\theta})$ for different training seeds in Figure S2. In our analysis we do not account for variations introduced by our surrogate model training process. However, Figure S2 suggests that these effects can be neglected in our case, as the standard deviation of the estimated correlation coefficient is about 1% for the different time points.

References

1. M. Klein, A. Sadiki, and J. Janicka. A digital filter based generation of inflow data for spatially developing direct numerical or large eddy simulations. *J. Comput. Phys.*, 186(2):652–665, April 2003.
2. J. Konrad, I.-G. Farcaş, B. Peherstorfer, A. Di Siena, F. Jenko, T. Neckel, and H.-J. Bungartz. Data-driven low-fidelity models for multi-fidelity Monte Carlo sampling in plasma micro-turbulence analysis. *Journal of Computational Physics*, 451:110898, 2022.
3. R. R. Linn. *A Transport Model for Prediction of Wildfire Behavior (No. LA-13334-T)*. PhD thesis, Los Alamos National Lab., NM (United States), 1997.
4. B. Peherstorfer, K. Willcox, and M. Gunzburger. Optimal model management for multifidelity Monte Carlo estimation. *SIAM Journal on Scientific Computing*, 38(5):A3163–A3194, 2016.
5. Q. Wang, M. Ihme, Y.-F. Chen, and J. Anderson. A tensorflow simulation framework for scientific computing of fluid flows on tensor processing units. *Computer Physics Communications*, 274:108292, 2022.

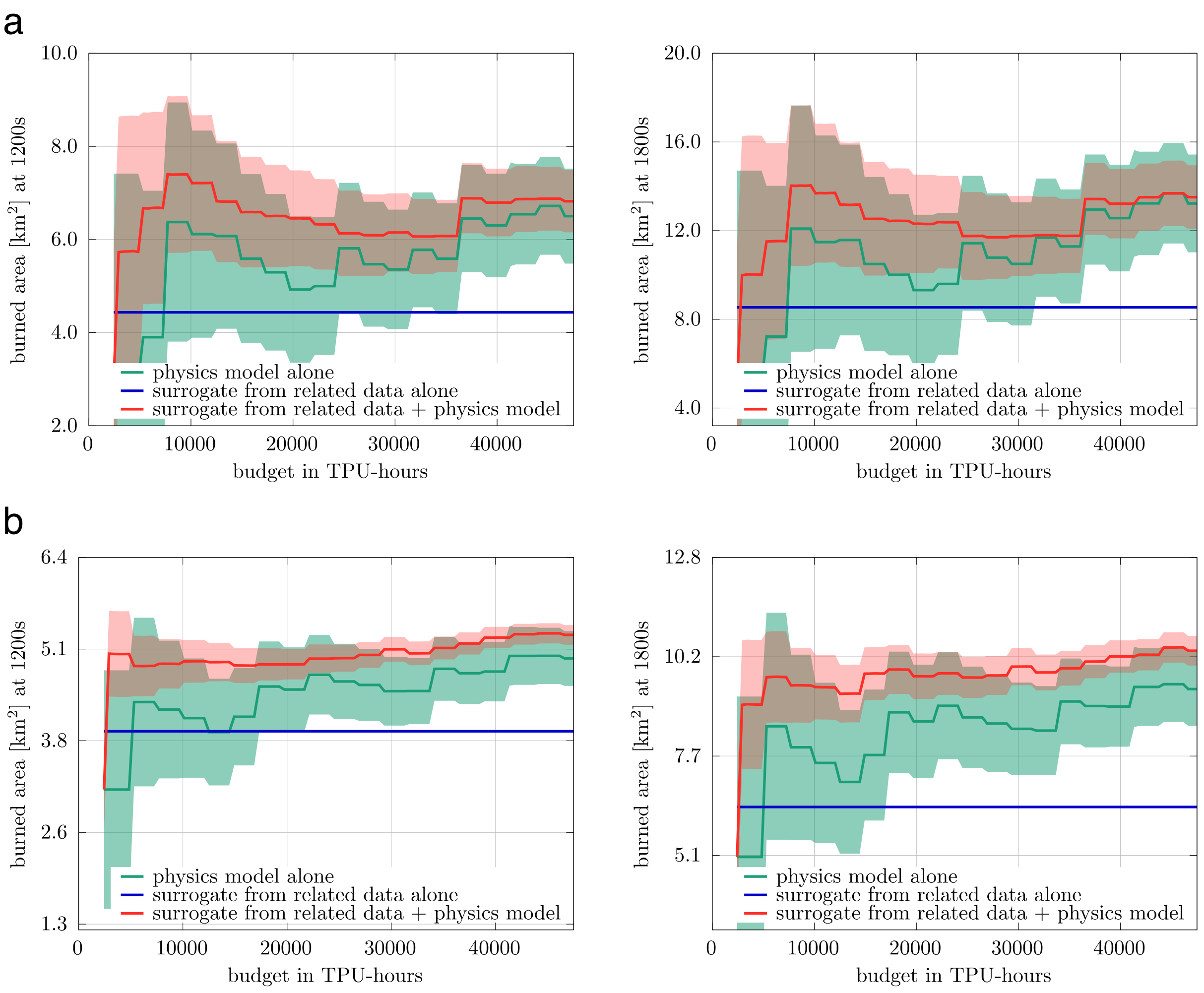

Figure S3. Comparison of different estimators at additional times after ignition. a: Scenario with input distribution (7), b: Scenario with input distribution (8).
